# Similar simulation of overburden movement characteristics under paste filling mining conditions

**DOI:** 10.1038/s41598-023-39782-3

**Published:** 2023-08-02

**Authors:** Pengfei Wu, Jihe Zhao, Jiaxu Jin

**Affiliations:** 1 Liaoning Technical University, Fuxin, 123000 China; 2 Key Laboratory of Geotechnical and Underground Engineering of Ministry of Education, Tongji University, Shanghai, 200000 China; 3 Liaoning Key Laboratory of Mine Subsidence Disaster Prevention and Control, Fuxin, 123000 China

**Keywords:** Mineralogy, Civil engineering

## Abstract

The method of filling mining can solve the problem of surface subsidence caused by coal mining. Among them, it is crucial to study the mechanism of filler strength improvement timeliness and filler mining to control rock movement for filler mining. In this paper, by combining theoretical analysis and similar simulation experiments, compressive strength is used as the research parameter to conduct proportioning test research on paste filling similar materials such as coal gangue, fly ash, and cement. The results prove that the strengths of the test ratios can meet the strength design criteria and lay the foundation for the requirements of similar simulation experiments. In order to study the characteristics of overburden failure, stress and displacement in the process of filling mining, the key technical parameters of overburden movement are determined. Similar simulation experiments were conducted to study the movement and deformation of overburden rock and the displacement and stress distribution law of overburden rock in the coal mine under different filling rates and filling steps conditions. The results show that the filling rate and filling step are the keys to preventing the overlying rock from rupture and collapse, and the larger the filling rate is, the smaller the stress and displacement of the overburden; the larger the filling step is, the larger the displacement and stress change of the overburden, and vice versa. In addition, the displacement curve along the strike is basically an "arch" type distribution, and the stress variation trend is "large-small-large" with a "Z" type distribution. The research results are of great significance to guide the practice of filling mining and can provide the theoretical basis for its further promotion.

## Introduction

It is estimated that the area of wasteland caused by mineral resources development in China is about 200 million mu, and this number is still increasing with the growth of coal production^1^. To completely solve a series of environmental problems such as surface subsidence and surface damage caused by coal mining, Academician Qian et al.^2^ proposed the concept of coal mine green mining technology (GMT) method at the beginning of the twenty-first century, and systematically expounded its technical framework, which pointed out the direction for solving mining damage and environmental problems. The green mining system^3–7^ includes water preservation mining, subsidence reduction mining, gangue reduction mining, coal and gas co-mining, and underground gasification mining, and it is noteworthy that the first three items all involve filling mining. Other scholars^8,9^ researched solid filling materials, processes, equipment, and overburden movement laws, and formed a systematic system of mining, beneficiation, and filling green mining technology. After long-term research and development, various scholars have formed coal mine paste filling mining technologies that can effectively control the movement and deformation of overlying rock formations and solve many environmental problems, which can achieve the purpose of replacing "three under" coal resources and preventing surface subsidence on the basis of controlling the movement and deformation of the overlying rock layer in the coal mining site^10,11^. Therefore, it is crucial to study the mechanism of filling mining to control rock movement for filling mining.

Many coal mine filling methods have been developed, including hydraulic filling method, direct filling method and paste filling method^12^. Among these filling methods paste filling is the most effective and has the advantages of high filling rate and high strength, which can form a filling body with certain compressive strength within 7 d, timely support the roof of the mining area and limit the movement deformation of the overlying rock layer. Xue et al.^13,14^ conducted triaxial compression and percolation tests on coal underground materials and analyzed the effect of gas pressure on their mechanical parameters. Wang et al.^15^ conducted uniaxial compression tests on paste-filled materials and proved that the preparation of paste-filled materials using coal-based solid wastes to fill underground mining areas is a feasible treatment method. However, since the compaction process of filler is particularly complex and the constitutive relation is constantly changing, while the selection and proportioning of materials will directly affect the physical and mechanical properties of the test model^16,17^. Therefore, how to simulate the filler strength to improve the timeliness and other issues remain to be further studied, which will have good economic benefits to society while maintaining the natural ecological environment of the mine.

Field tests have the advantage of being realistic and reliable, but they are expensive and time-consuming, and it is difficult to grasp the main factors for mechanistic analysis. In particular, some destructive tests, and advanced foresight analysis cannot be carried out on-site, and it is difficult to directly observe the overburden stress change and damage characteristics data. Therefore, similar simulation experiment is an effective method to study the practical problems of mining engineering and is an important means and effective method to study the deformation and damage of overburden rock, stress distribution, and surface movement of coal mining. Zeng et al.^18^ studied the characteristics of overburden motion and stress evolution and designed a support scheme by combining similar simulation experiments in different engineering contexts. Jun et al.^19^ studied the stress, deformation, and fracture field characteristics of the roof and floor strata due to mining, an indoor large-scale three-dimensional physical similarity model was developed, and a novel method was used to simulate the mining process of coal seams. Ning et al.^20^ studied the rationality of mining and filling sequence by theoretical analysis and similar simulation, and revealed the stress and surface subsidence law of the filling body (or coal column) under two filling sequences. Yin et al.^21^ analyzed the effect of strength parameters of the filling body on the overburden movement and support pressure by numerical simulation method, and the study is a good guide for coal mine longwall working face paste filling.

The procedure of filling coal mines immediately after mining is always present, regardless of the paste filling material used, i.e., it needs to be filled before the overburden collapses after the extraction of the quarry. Only in this way can we have the effect of filling the mining space to the maximum and controlling the movement of the overburden^22^. The above-mentioned research results have deeply studied the mechanical characteristics and principles of rock movement control in filling mining from different perspectives, which have promoted the development and application of paste filling mining. However, the existing research results are less involved in the parameters of paste filling effect and process, which are not only related to the problems of filling material saving and filling cost, but also closely related to the overburden damage and surface movement deformation. Therefore, it is necessary to understand the relationship between the parameters of coal seam mining thickness, filling rate, and filling step that are related to the effect of overburden movement control. On this basis, this paper takes a coal mine in Shanxi Province as the research object, and conducts similar simulation experiments by filling the mined-out area. The study of the damage pattern of overburden rock in the mining-filling process under different parameter conditions and the influence of overburden rock displacement and support pressure in the advancing process is of great theoretical and engineering significance for coal mine paste filling mining.

## Strength proportioning design of similar materials for paste filling

### Theoretical strength design of cemented paste backfill

**Figure 1:**
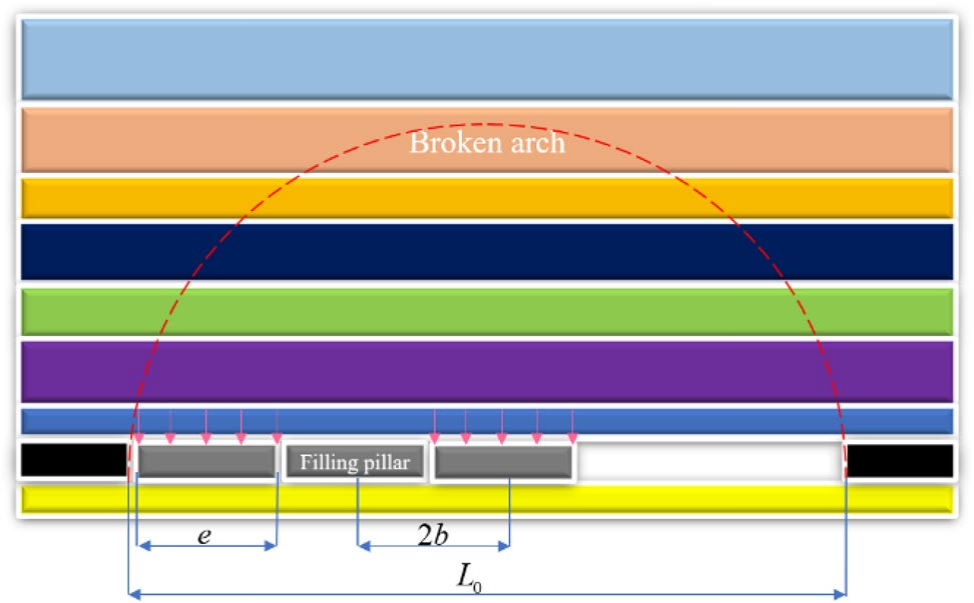
Structural mechanics model for coal mining.

According to the drilling and physical exploration data, a coal mine in Shanxi Province is located in the Qinshui coalfield, with a coal seam burial depth of about 100–150 m and a designed production capacity of 7.5Mt/a. About 372 million tons of coal pressed in the "three down" mines. Currently, the mine uses the paste filling method to mine coal, and the filling cycle step is two mining and one filling. The coal seam within the recovery area is a medium-thick coal seam, and its top and bottom plate parameters are shown in Table 1 below. On the basis of the analysis performed on the coal mine rock structure, the overlying rock layers were divided according to the key layer theory^23^. The key layer mainly consists of yellow clay, sand and gravel, medium-grained sandstone, fine-grained sandstone, siltstone, and coal seam. The total length of the mining workings was determined to be 98m, taking into account the variation of the fugacity state of the main key layer of the overlying rock layer and the error of the estimated fracture distance, and considering the coal mining efficiency. Assuming that the mineral pressure is uniformly distributed on the filling body, the structural mechanics model of the quarry is shown in Fig. 1. The rock weight inside the fissure arch can be supported by the filling body, which is necessary to ensure the stability of the filling zone, while the coal (rock) body around the quarry supports the rock weight outside the fissure arch.

**Table 1:** Table of roof and floor rock parameters.

Lithology	Thickness/m	Compressive strength/MPa	Tensile strength/MPa	Elastic modulus/GPa	Internal friction angle/°
Silt sandstone	4.175	46.77	5.1	15.69	28
Coal seam	5.14	10.05	1.7	6.31	20
Silt sandstone	7.79	31.08	3.3	12.74	32

The structural mechanics equation is:

1 $$\frac{1}{2}\pi \left( {\frac{{L_{0} }}{2}} \right)^{2} C_{x} \gamma = \frac{e}{2b}L_{0} C_{x} q \cdot 100$$

where $L_{0}$ is the length of the working face, m; $C_{x}$ is the working face advance distance, m; $\gamma$ is the bulk weight of the rock formation, t/m^3^; $e$ is the filling step, m; $2b$ is the filling body spacing, m; $q$ is the uniform stress distribution load in the filling zone, MPa.

From Eq. 1, the uniform load of paste filling is:

2 $$q = \frac{{\pi L_{0} b\gamma }}{400e}$$

Therefore, the design criterion for the strength of the filling body of a single working face mining is:

3 $$a = \lambda \frac{{\pi L_{0} b\gamma }}{400e}$$

where, $a$ is the filling strength, MPa; $\lambda$ is a safety factor.

In this paper, the length of the working face of single quarry is 98m, i.e. $L_{0}$ = 98 m; the coal seam capacity is $\gamma$ = 1.4 t/m^3^; the filling step is *b* = $e$ = 2 m; $\lambda$ = 1.5 (safety factor), resulting in the theoretical design strength of the filling body $a$ = 1.62 MPa.

### Preparation of similar materials for paste filling

At present, there is no relevant test procedure standard for paste filling, so the material preparation for this test mainly refers to the Standard for Basic Performance Test Methods of Mortar (JGJ/T70-2009)^24^ and the Standard for Performance Test Methods of Ordinary Concrete Mixes (GB/T50080-2016)^25^.

Coal mine filling paste is an ideal material with good fluidity, uniform texture, and low water consumption. Experiment using coal gangue as the main aggregate, its rich sources, easy to take materials, low price, while reducing the harm caused by the gangue piled on the ground. Using the vibrating sieve machine gradation sieving preparation of particle size less than 25mm filling aggregate, its particle size in line with the "gravel or pebbles of the particle gradation range" provisions, good gradation. Cement and gypsum are used as cementitious materials to improve stability, and have a very important impact on the physical and mechanical properties of the cement before and after solidification. The cement is ordinary silicate cement (code P-O), the initial setting time shall not be less than 45 min, the final setting shall not be greater than 600min, and the water-cement ratio is 0.5. The relative density of gypsum is 2.96 g/cm^3^, the initial setting time shall not be less than 6 min, and the final setting shall not be greater than 30 min. This test uses water-reducing agent, expansion agent, and fly ash as an admixture to improve the performance of the paste filling body. Using fly ash as an additive can improve the compatibility of the paste, reduce the amount of cement, and at the same time put forward a new method and new measures for the comprehensive utilization of the existing solid waste in China, effectively solving the problem of land resources and environmental pollution brought by fly ash, which is a win–win, efficient, valuable and good additive material for the paste filling body.

**Figure 2:**
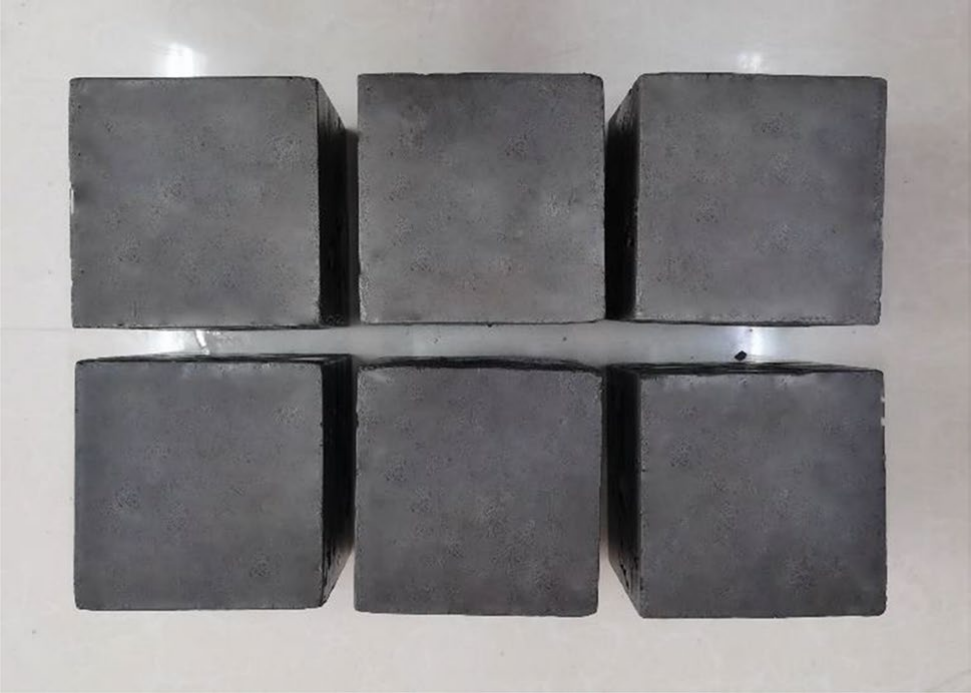
Cemented paste backfill specimen.

Since material proportioning has a great influence on the physical and mechanical properties (especially the material strength) of the cemented paste backfill^26^, it is important to study the strength of paste filling materials. In this experiment, a single variable control comparison method was used for five groups of coal mine paste filling materials with different proportioning schemes, and the contents of fly ash, gypsum, cement, and water-reducing agent have increased appropriately under the premise that the gangue content, expansion agent content, and water addition were kept constant. Figure 2 shows the paste filling test block prepared by manual mixing to meet the requirements, and its main ratios are shown in Table 2.

**Table 2:** Cemented paste backfill test material proportioning table.

Groups	Factor level				
	Fly ash: coal gangue	Gypsum: coal gangue	Cement: coal gangue	Water reducer: cement, gypsum	Expansive agent: cement
Ratio 1	3:5	1:10	4:25	1:13	7:8
Ratio 2	5:5	1:10	4:25	1:13	7:8
Ratio 3	3:5	2:10	4:25	1:13	7:8
Ratio 4	3:5	1:10	6:25	1:13	7:8
Ratio 5	3:5	1:10	4:25	2:13	7:8

### Compressive strength analysis of paste filling materials

Evaluating the compressive strength characteristics of the cemented paste backfill under different proportioning schemes and selecting the optimal proportioning scheme can accumulate experience for the development of coal mine paste filling technology and promote the development of the coal mine-filling technology industry. After filling, the roof plate of the mined-out area can be greatly supported by the paste body, which has the function of limiting the ground settlement. The strength of the paste filling body has an extremely important position in the whole process of production use and later operation management, and its strength is directly related to the stability of the whole filling area. Therefore, the compressive strength index of the paste fill is an indispensable mechanical parameter in geotechnical engineering research, design, construction, and production^27^.

**Figure 3:**
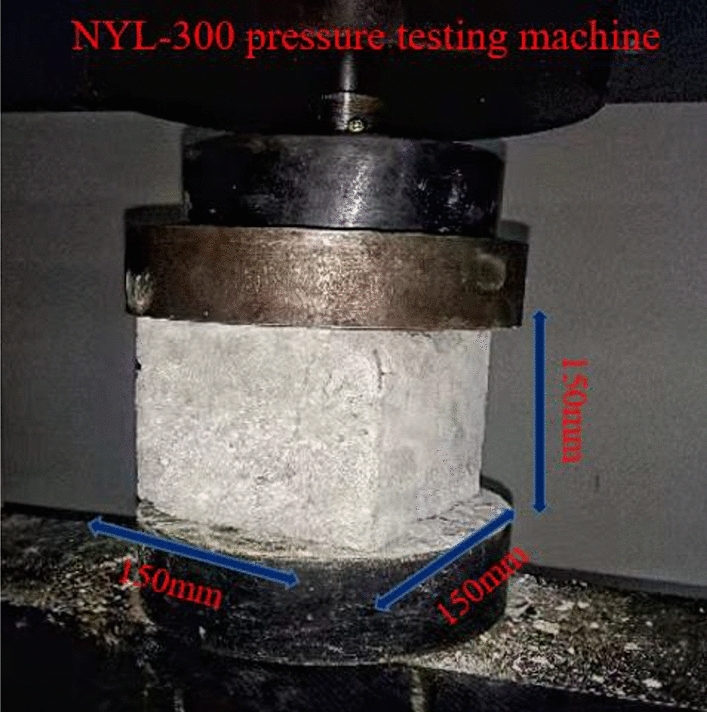
Uniaxial compressive test chart.

**Figure 4:**
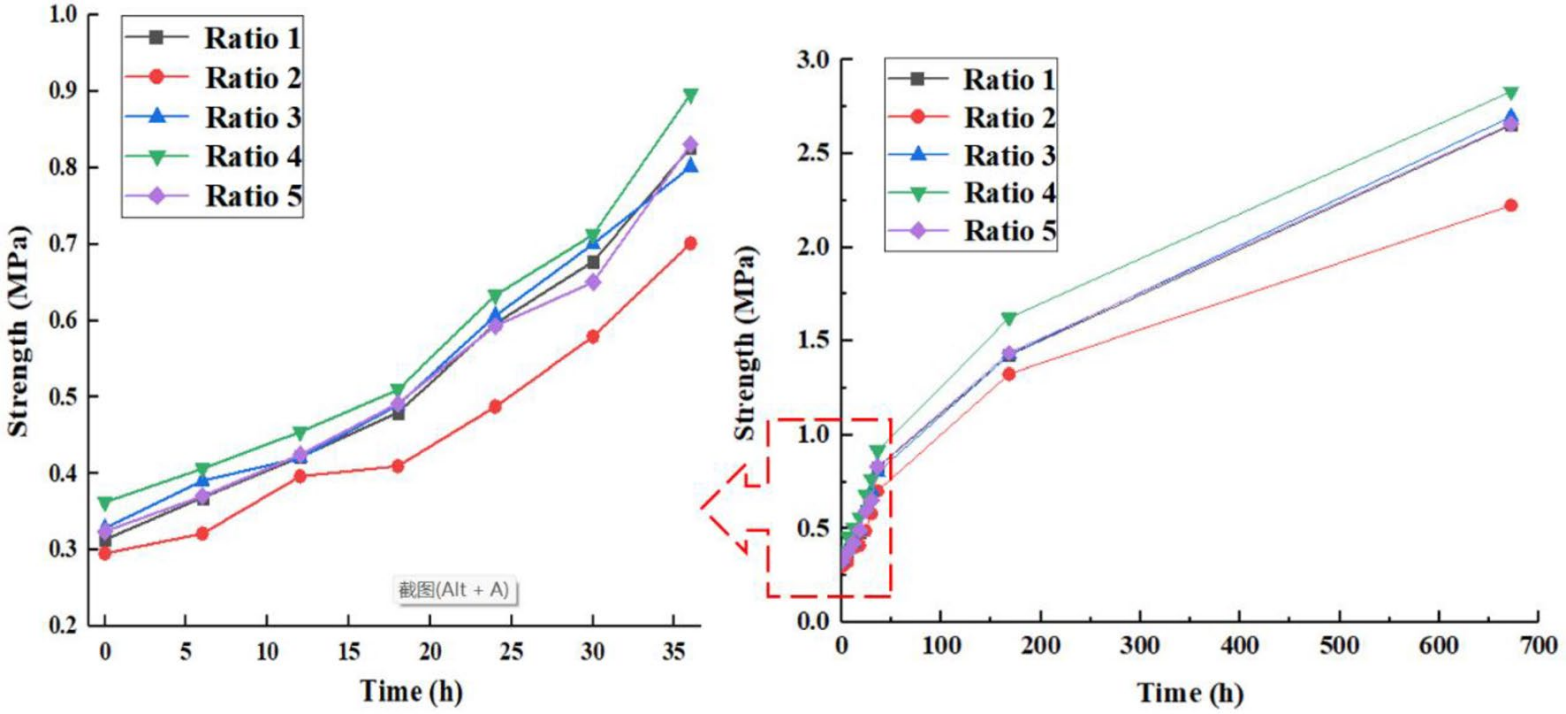
Comparison curve of strength of different proportioned pastes.

**Figure 5:**
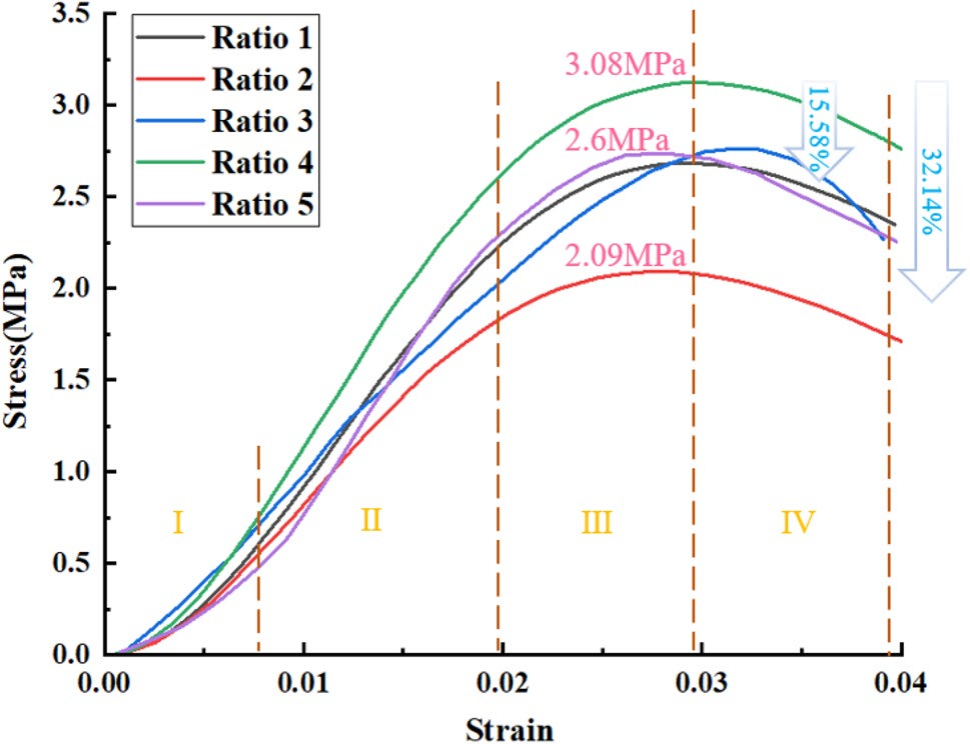
Stress–strain curves of different proportions of paste.

The compressive strength properties of the paste in the mined-out area are crucial because it is mainly subjected to the pressure of the overlying rock layer^28^. In this test, the specimens were created using a removable mold with dimensions of 150 mm × 150 mm × 150 mm, and after removal from the mold, the specimens were placed in a YH-40B standard constant temperature (curing temperature 22 °C) and humidity (85%) curing chamber for curing. The specimens shown in Fig. 3 were tested for uniaxial compressive strength using NYL-300 universal material pressure tester with a maximum load of 300 kN and 0.1% accuracy, and the loading rate was taken as 0.3 MPa per second. To facilitate the observation of the variation pattern of early intensity it is plotted as a curve as shown in Fig. 4. The uniaxial compressive strength is an important parameter to measure the quality of the filling body. Figure 5 shows the stress–strain curves of five different ratios of paste fillers during uniaxial compression. The curves are divided into compaction stage (I), elastic deformation stage (II), plastic deformation stage (III) and post-peak damage stage (IV)^29^. The strengths of ratios 1 and 2 were reduced by 15.58% and 32.14%, respectively, compared to ratio 4. The paste-filled bodies of each ratio in stages I and II exhibited similar stress–strain phenomena, and the curves basically overlapped, indicating that the filled bodies experienced the same structural changes in this stage. In stages III and IV, the paste-filled specimens gradually underwent plastic damage, and the curves showed different expressions due to different ratios.

According to the observation of Fig. 4, it can be seen that the strength of the filled body will gradually increase with time, with late strength changes being more noticeable than early strength changes. In comparison to other ratio schemes, the strength of ratio 4 is the strongest, with a 28 d strength that can reach 2.832 MPa; the strength of ratio 2 is the weakest, with a 28d strength that is only 2.224 MPa. Further analysis shows that too much fly ash will reduce the strength of the paste. Due to the small fineness of fly ash and close to the fineness of the paste aggregate, it can also act as an aggregate component in the paste, so the promotion effect of fly ash on the paste filling material is somewhat limited. This is the primary cause of its inability to raise the paste's strength index^30^. Due to its higher cement concentration, Formulation 4 ranked highest in both early and late strength, proving that cement is the primary component influencing the paste's strength. Because of aggregate's particle size factor, its contribution to strength is constrained. The strength of the paste is mainly determined by the properties of the gelling material, thus it stands to reason that this substance is also a factor in determining the paste's strength.

In the process of using paste filling in coal mines, the faster the filling material reaches the theoretical design strength, the faster the construction progress can be accelerated and the safety risks caused by overburden settlement can be reduced. Ratio 4 can reach the theoretical design strength (1.62 MPa) at 7 d, and its strength is always higher than other ratio options. Therefore, Ratio 4 was selected as the optimal ratio of paste filling material.

## Simulation tests of similar materials for paste filling

Model tests are one of the main research methods in geotechnical engineering. Compared with field tests, laboratory model tests need to simplify the initial conditions and boundary conditions, and there are certain errors, but considering that field tests are more expensive and time-consuming, especially some destructive tests and over-anticipatory analysis, which cannot be performed on-site. Therefore, it is necessary to conduct model tests.

### Establishment of mechanical model and analysis of filling parameters

**Figure 6:**
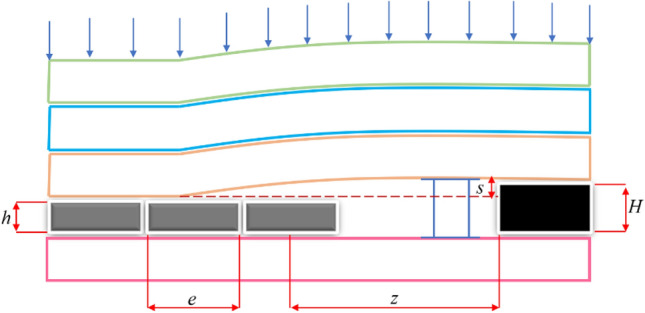
Spatial relationship model of "surrounding rock-filling body-support".

The structural changes of overburden rock caused by the autonomous movement of coal mine rock make it different from the rock mechanics model of conventional quarry filling. Figure 6 establishes the overall mechanical model of the filling quarry^21^, and the main parts of the model are overburden rock and filling body. The filling model includes the following parts: ① Overburden rock: due to the limited space for overburden movement, the overburden basically does not form a rock beam structure; ② Filling body: sinking of the overburden can be well controlled; ③ Support: relatively gentle changes in the filling quarry support load, the cycle comes pressure is not obvious; ④ Floor: bottom bulge at the bottom of the filling quarry is less than in a normal quarry.

The control of overburden movement is mainly determined by the filling effect, the filling process and the interaction between them. The filling effect mainly refers to the filling rate of the quarry. As the filling rate increases, the overburden movement space becomes smaller, i.e. the displacement will be reduced. The filling process mainly refers to the filling step. The larger the filling step, the more time the overburden sinks, the larger the sinkage, the filling rate of the mined-out area will be reduced. In order to ensure efficient production, it is important to choose the appropriate filling step based on geological conditions. It should be noted that if the filling step becomes smaller, the number of filling times will increase, which may not be beneficial for production The filling cycle step is generally "one mining and one filling, two mining and one filling, three mining and one filling", rarely "four mining and one filling" or even a larger filling step, too large filling step has no value for research^31^.

From the above analysis, it can be seen that the main parameters of overburden movement control in coal mine filling mining are filling rate and filling step. The relationship of the control parameters is derived from the overall rock mechanics model of " surrounding rock-filling body-support " in the filling mining site.

4 $$H = s + h$$

5 $$n = h/H$$

6 $$z = Ke(K \ge 1)$$

7 $$w = s + fh$$

where $H$ is the thickness of the coal seam in the unit of m; $h$ is the filling height in the unit of m; $s$ is the unfilled height in the unit of m; $n$ is the filling rate, $z$ is the distance between the location of the overburden rock contacting the filling body and the coal wall in the unit of m; $e$ is the filling step in the unit of m; $K$ is the filling coefficient; $w$ is the final settlement value in the unit of m; $f$ is the filling deformation coefficient.

### Similar simulation experimental scheme

#### Similar parameters determination

The essence of similarity simulation is to use materials with similar mechanical properties to the prototype and scale them by similar constants to make a model. The test is conducted by simulating excavation so as to observe and study the deformation and damage of overburden rock and other phenomena. According to the three experimental theorems of geometric similarity, kinematic similarity and dynamic similarity of similar test principles^32^, the design geometric similarity ratio $C_{L}$ is the ratio of the prototype size $L_{H}$ to the model size $L_{M}$, i.e.,$C_{L} = L_{H} /L_{M} = 1:100$. The density similarity ratio $C_{\gamma }$ is the ratio of the prototype apparent density $\gamma_{H}$ to the model apparent density $\gamma_{H}$, i.e., $C_{\gamma } = \gamma_{H} /\gamma_{H} = 1:1.8$. The stress similarity ratio $C_{\sigma }$ is the product of the density similarity ratio $C_{L}$ and the geometric similarity ratio $C_{\gamma }$, i.e., $C_{\sigma } = C_{L} /C_{\gamma } = 1:180$. The time similarity ratio $C_{t} = \sqrt {C_{L} } = 1:10$. According to the geometric similarity ratio simulation test model length × width × height is $0.98{\text{m}} \times 0.3{\text{m}} \times 0.84{\text{m}}$.

#### Displacement and stress measurement point arrangement

**Figure 7:**
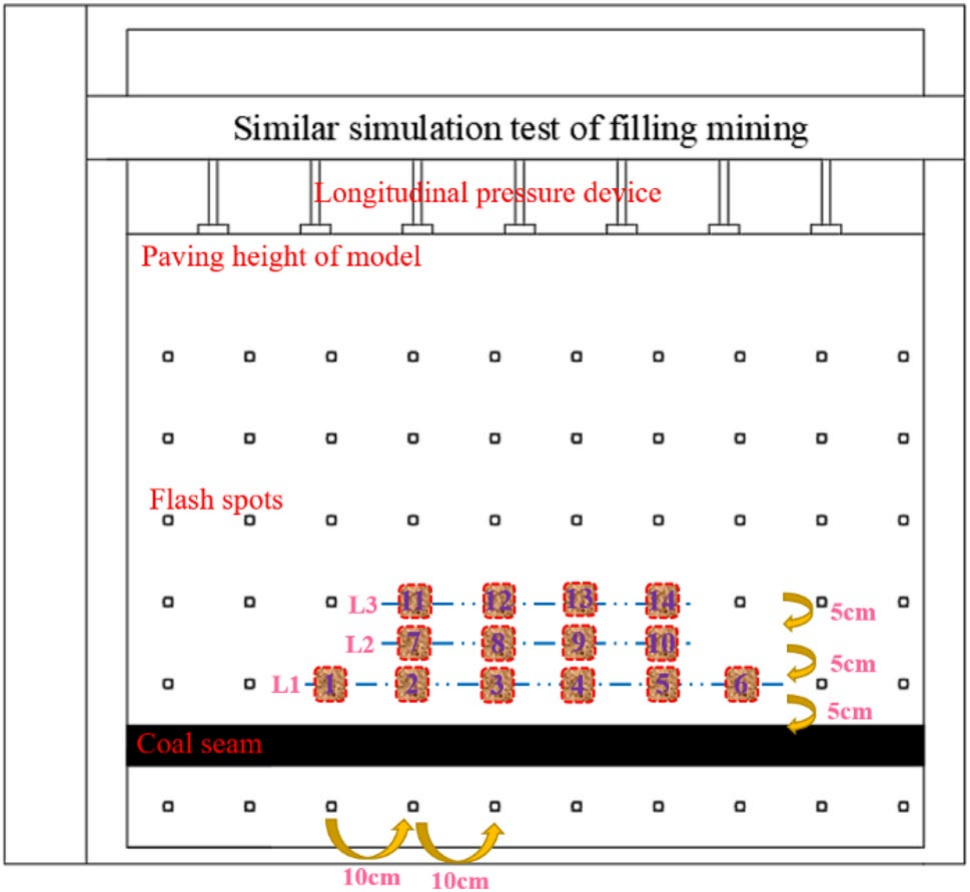
Physical model of coal mine paste mining.

Similar simulation experiments of filling mining in the similar simulation experiment box were conducted in the similar simulation box^33^. There are three displacement measurement lines as shown in Fig. 7, which are located 5, 10, and 15 cm above the coal seam, and the measurement lines are L1, L2, and L3 in order from bottom to top. for the rock with more developed overlying joints, the model is laid in layers. Mica was added in the middle of the model to simulate the overlying joints. The stress sensing equipment used for the arrangement of the measurement points was a circular stress box of 36 mm thickness and 15 mm radius, and the XJTUDP 3D optical photogrammetry system was used to observe the amount of change in displacement of the overlying rock layer up to the surface measurement point during the mining and charging process, and then analyze the movement change law of the overlying rock^34^.

#### Similar model materials and filling scheme setting

The rock layers in the similarity simulation experiments were mainly made by using quartz sand, gypsum, and lime as similar materials in proportion to their strength. Table 3 shows the parameters of similar materials calculated according to the similarity criterion and the similarity ratio of the model. After the actual mining seam is extracted according to the two mining and one filling method, that is, the filling is carried out according to the filling step of 2m. According to the field practice, the filling rate of coal mined-out area generally reaches 80–95% to effectively control the overburden movement. In order to investigate the effect of different filling processes and filling effects on the overburden control, the influence of mine pressure and the economics of safe production in coal mine paste filling mining, therefore, by using different mining-filling steps (2cm, 3cm) and different filling rates (85%, 95%) for the test, the changes in displacement and stress during the mining-filling process of coal mine paste were observed to provide a theoretical basis for the efficient and safe mining of mineral resources^35^.

**Table 3:** Similar model coal rock material parameter.

Lithology	Model thickness/cm	Model volume/m3	Total mass of model/Kg	Sand/Kg	Lime/Kg	Gypsum /Kg	Water/Kg
Yellow clay	40		794.1	697	67.6	28.9	88.2
40 layers	1	0.003	4.95	4.33	0.43	0.18	0.55
Sand gravel	10.41		91.5	76.3	7.6	7.6	10.2
1 layer	1.8	0.005	8.25	6.88	0.68	0.68	0.91
5 layers	1	0.003	4.95	4.12	0.41	0.41	0.55
1 layer	1.71	0.005	8.25	7.07	0.35	0.825	0.91
1 layer	1.90	0.0055	9.07	7.93	0.56	0.56	1.0
Medium-grained sandstone	8.05		94.5	81	4.1	9.5	10.5
7 layers	1	0.003	4.95	4.24	0.21	0.49	0.55
1 layer	1.05	0.003	4.95	4.33	0.31	0.31	0.55
Fine-grained sandstone	7.0		94.5	81	4.1	9.5	10.5
7 layers	1	0.003	4.95	4.24	0.21	0.49	0.55
Rock siltstone	4.175		34.5	30.1	1.3	3.0	3.8
1 layer	1.54	0.0045	7.43	6.5	0.28	0.65	0.82
1 layer	1	0.003	4.95	4.3	0.18	0.43	0.55
1 layer	1.635	0.005	8.25	7.2	0.51	0.51	0.91
Coal seam	5.14	0.015	24.75	22	1.92	0.82	2.75
Rock siltstone	7.79		105	91.8	3.93	9.18	11.6
1 layer	1.79	0.005	8.25	7.2	0.31	0.72	0.91
6 layers	1	0.003	4.95	4.3	0.18	0.43	0.55

### Analysis of similar simulation results of paste filling

#### Characteristics of broken ring of overburden under non-filling conditions

**Figure 8:**
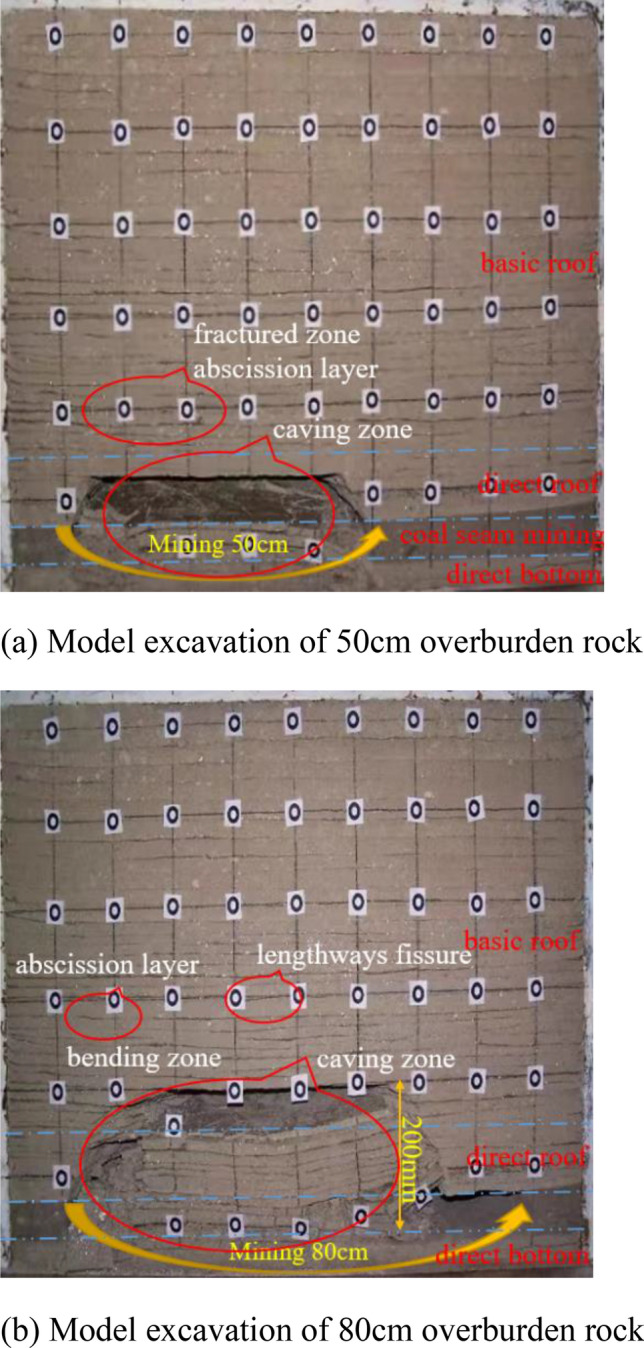
Physical model of similar test for caving mining.

In collapse method mining, as the working face advances, the overlying rock layer bends and sinks as a whole, and will produce the collapse and breakage of direct top and basic top, and the overlying rock has obvious characteristics of three belts (collapse belt, fracture belt and bending and sinking belt). As shown in Fig. 8, when the working face is advanced to 50 cm, the basic roof continues to expand upward and collapse, and the overlying rock layer behind the working face bends and sinks, gradually approaching the coal seam floor to form a stable structure. When the excavation reached 80cm, the model overburden was destabilized and the collapse height exceeded 20 cm, the overburden damage on the working face developed to the maximum extent, meanwhile, the direct top of the working face and the basic top off layer at the back of the working face basically closed and touched the coal seam floor, and the overburden structure remained stable.

**Figure 9:**
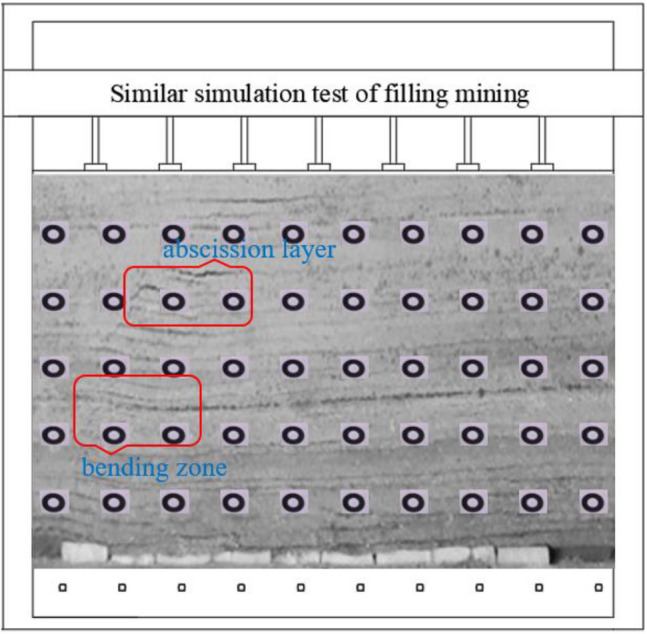
Similar test model of filling mining.

The filling process in Fig. 9 is realized by using filling blocks made in advance and stuffed into the post-mining void. Compared with the collapse method, the presence of the filling body can effectively support the overlying rock layer, and its bending settlement is smaller and collapse will not occur. The presence of the filler acts as a coal pillar, which is not fully equivalent to the action of the coal pillar due to the limited strength of the filler. And there is a problem with filling rate, filling can not completely limit the occurrence of phenomena such as cross top, but the existence of filling body will greatly improve the overburden stability. The top plate only appears parallel to the laminae fractures in the lower rock layer, with no penetrating fractures in the vertical direction, and no fracture occurs in the direct top. As the working surface continues to advance, the rock layer above the basic top will be separated at the interface, the fissures below are in a closed state, and the overburden structure as a whole presents a stable state.

#### Overburden movement law under paste filling conditions

When the filling rate and filling step vary, there are large differences in the displacement of different rock formations^36^. Table 4 shows the maximum displacements measured by controlled experiments for 2 cm filling and mining step filling rate 85% (case 1), 2 cm filling and mining step filling rate 95% (case 2), 3 cm filling and mining step filling rate 85% (case 3), and 3 cm filling and mining step filling rate 95% (case 4).

**Table 4:** Maximum subsidence displacement of overburden for each working condition.

Displacement	Survey line											
	Condition 1			Condition 2			Condition 3			Condition 4		
	L1	L2	L3	L1	L2	L3	L1	L2	L3	L1	L2	L3
20 cm maximum displacement (mm)	3.9	3.6	3.1	1.8	1.6	1.3	4.4	4.0	3.7	2.0	1.7	1.5
40 cm maximum displacement (mm)	5.6	5.1	4.6	2.0	1.8	1.6	6.1	5.7	5.2	2.2	1.9	1.7
60 cm maximum displacement (mm)	6.3	5.9	5.1	2.2	2.1	2.0	7.0	6.8	6.0	2.4	2.3	2.2

**Figure 10:**
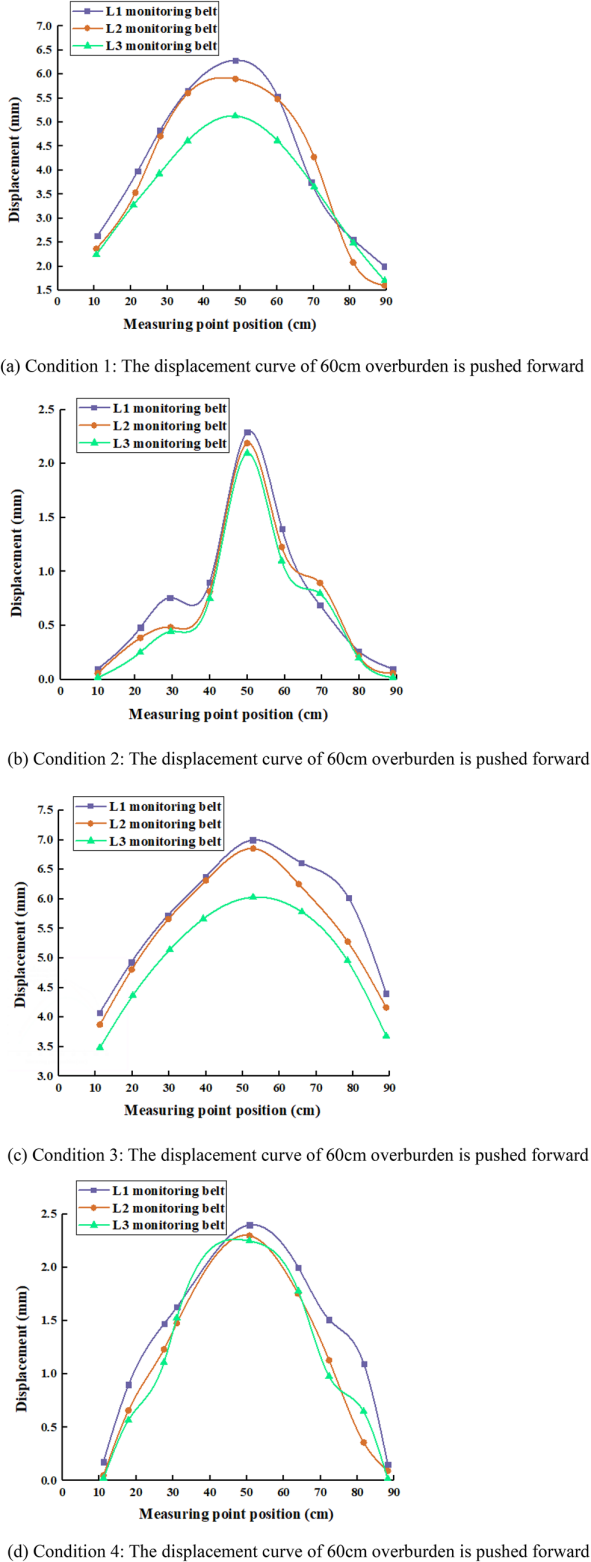
Distribution of overburden displacement curve.

Figure 10 shows the analysis of the distribution law of overburden displacement when the working face is advanced 60cm under different working conditions, and the rock displacement curve basically shows "arch" type distribution characteristics. The arch top is basically located at the center of the workface advance length, that is, the farther away from the start mining line and the workface, the greater the sinking amount. The direct top (L1 measuring line) has the largest sinkage, and the L3 measuring line has the smallest displacement, i.e. the further away from the working face in the vertical direction, the smaller the displacement, and the displacement between measuring lines of different heights is obviously distinguished, but the difference is small. Because the rock layer above the basic top is developed in the form of off-layer when filling mining, when the rock movement tends to be stable, the compaction between each rock layer is better, so the sinking amount does not change much.

Analysis of the overburden movement law with different filling rates (working conditions 1 and 2, working conditions 3 and 4 for comparison). The increasing values between the locations of each point are basically the same as the mining proceeds. As the filling rate of the filling body increases, the displacement shrinks with the increase of the advancing distance, the displacement space of the mined-out area decreases, the overburden fissure development height decreases, and the place where collapse will occur is effectively limited. Increasing the filling rate has a better contribution of limiting displacement throughout the mining process^37^.

Analyzing the overburden movement pattern at different filling steps (working conditions 1 and 3, working conditions 2 and 4 for comparison), the displacement is more stable at smaller filling steps. Although the trend of the curve remains the same, the displacement of the working face also becomes larger due to the larger filling step. Therefore, too large a filling step will have a very negative impact on the filling, so try to choose a smaller filling step.

#### Overburden stress evolution distribution law under paste filling conditions

In the process of working face advancement, the support pressure in front of the working face will increase and a pressurization zone will appear in a certain range; the support pressure behind the working face will decrease and a decompression zone will appear in a certain range; outside the range of mining influence, the distribution of stress tends to be similar to the original rock stress. The distribution range and characteristics of the pressurization and decompression zones, i.e. the distribution of the support pressure at the working face, are closely related to the mining method, filling rate, and filling step.

**Figure 11:**
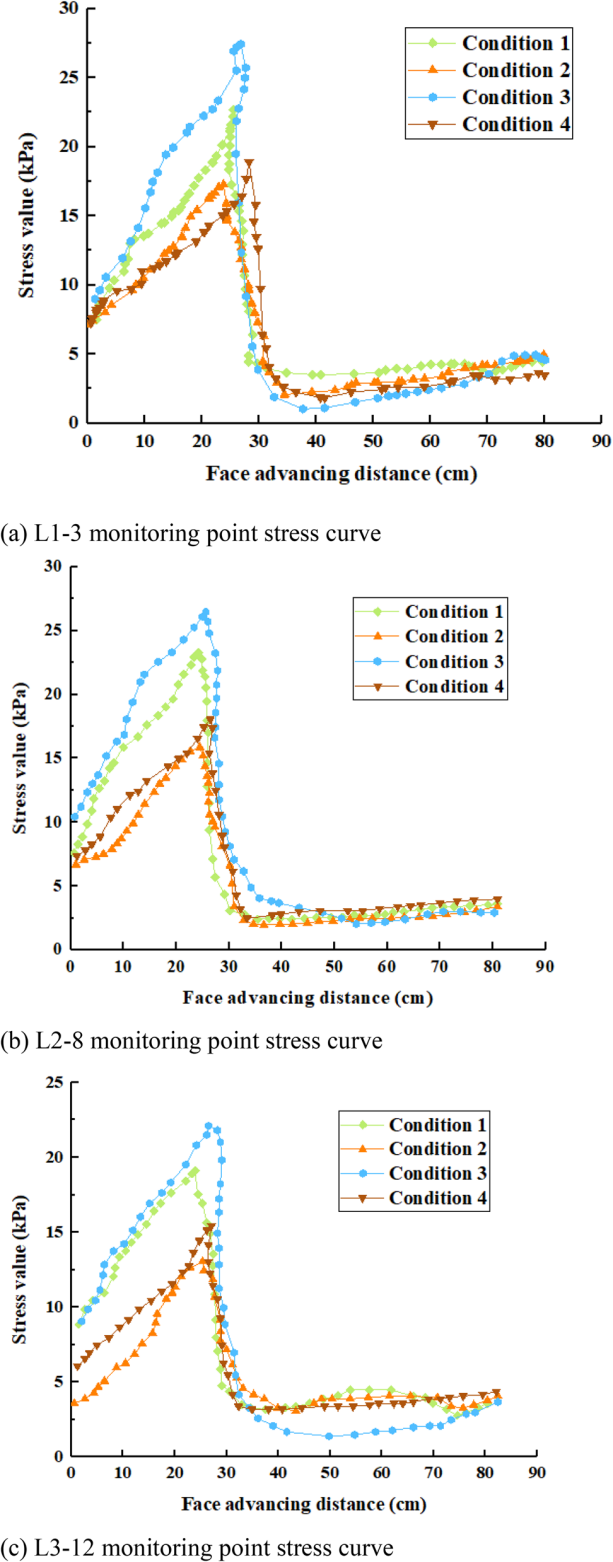
Stress variation curves for different working conditions.

In the process of working face advancement, the stress measurement point data located at the middle position of the roof plate reflects the evolution law of the roof plate stress with the advancement of the working face, i.e. the location of the measurement point with larger stress value appears. Therefore, the stress data of measurement points 3, 8, and 12 (code: L1–3, L2–8, L3–12) are listed, i.e. the further away from the working face in vertical direction, the smaller the stress value is. Although there are obvious distinctions in overburden stress under different working conditions, the trend of stress evolution distribution is basically the same. It can be seen from Fig. 11 that when the working face advances from the open-cut eye, the measurement point shows the original rock stress. As the working face advances in the direction of the measurement point (0–20 cm), the top plate stress gradually increases, and when the working face advances below the measurement point (20–30 cm), the stress rapidly decreases, which indicates that the rock layer appears to be delaminated. The stress of the overlying rocks gradually approached the original rock stress and stabilized after the working face was mined, which indicates that the bending and sinking of the rock layers began to touch each other. As mining continues, the delamination continues to develop upward. With the advancement of the working face, the trend of stress change is "big-small-big"^38^, and it shows the characteristic of "Z" type distribution. The filling mining does not change the stress state in front of the coal wall but only changes the magnitude of the stress, and the stress in front of the coal wall is partly shared by the filling body.

The overburden stress evolution law is analyzed for different filling rates (working conditions 1 and 2, working conditions 3 and 4 for comparison). Due to the increase of filling rate, the relative height of the mined-out area decreases, the damage degree of the roof plate decreases and the bearing capacity increases, the peak stress in front of the coal wall decreases significantly compared with the filling rate of 85%, the stress concentration coefficient decreases, the area of the pressurization zone is reduced and the influence range becomes smaller. As the filling rate increases, the height of the mined-out area becomes smaller, the filling body also shares part of the load of the roof rock layer, and the scope of the stress decompression zone at the back of the working face becomes smaller. Therefore, increasing the filling rate can limit the sinking and collapse of the roof slab, reduce the transfer of stress from the roof slab to the working face and the support pressure of stent, and reduce the risk of coal wall flaking.

The overburden stress evolution law is analyzed for different filling steps (working conditions 1 and 3, working conditions 2 and 4 are compared). The larger the filling step, the further the peak stress in front of the working face is from the coal wall and the larger the value, but the magnitude of change is more stable and the curve trend is basically consistent. The distribution law of stress at the back of the working face is exactly the opposite, which is not obvious but can be faintly visible. Therefore, the filling step is too large, which will have a very negative impact on the filling, so try to choose a smaller filling step.

## Discussion

At present, in the actual engineering field, there are disadvantages of paste filling materials such as low initial strength and easy to plug pipes, which affect the backfill effect and benefit. Therefore, we use compressive strength as the index to select the optimal ratio of similar materials for paste filling in the experiment. Through a combination of theoretical analysis and similar tests, we simulate the displacement law and stress evolution characteristics of the overburden under different filling rates and different filling step parameters. The reasonable optimization of paste filling control parameters has a good effect on ensuring the stability of overburden rock, which is consistent with the results of previous studies on filling and mining control parameters^18,20^. The limitation of this study is that the mechanical properties of paste filling materials are not considered enough and the research aspect is rather single, thus the paste similar effect is slightly different from the engineering reality. With the depletion of shallow buried coal resources, there is a development trend of coal seam mining to deep underground. Deep coal mining faces threats such as high ground stress and mining disturbance^39,40^. Paste-filled mining can effectively alleviate the degree of rock behavior, reduce the coal-rock power disaster, and is suitable for long-distance material transportation, which is very favorable for deep mining. Therefore, it is necessary to explore the new mode of paste backfill suitable for deep underground environment in the future. The future intelligent coal mining will be a deep integration of modern science and technology with coal mining technology. The filling material can be selected in the filling intelligent dosing system, and the filling process can be verified by intelligent software to verify the accuracy and scientific rationality of the overburden movement law of the paste filling conditions. The research results are expected to provide practical information for the work of coal mine filling and mining technology.

## Conclusions

Research shows that the roof of the mined-out area can greatly improve the bearing capacity under the support of the paste, which has the effect of limiting the ground settlement. Different ratios have a great influence on the strength of the filling body, and it is most critical to study the time-effectiveness of the filling body strength improvement. A more reasonable filling parameter was selected through similar simulation experiments, and the displacement law and stress evolution characteristics of the overburden under the filling parameter were analyzed. The specific conclusions are as follows.

1. When the theoretical design strength of the paste filler reaches 1.62 MPa, the paste filler can safely support the overburden weight and effectively control the surface subsidence, as obtained by the coal mining mechanics model. A kind of paste filling material is obtained to meet the similar strength: fly ash: gangue (3:5), gypsum: gangue (1:10), cement: gangue (6:25), water reducer: cement and gypsum (1:13), and expander: cement (7:8).
2. The overall mechanical model of "surrounding rock-fill body-support" in the infill mining site is established and the relationship between the control parameters is derived, and the analysis shows that the overburden movement control is mainly determined by the filling effect, filling process, and the interaction between them. In the process of coal mine paste filling, various means should be taken to increase the filling rate of the quarry and to choose the smallest possible filling step. The optimization of both will have a better effect on ensuring the stability of the overburden.
3. The filling rate and filling step have a significant effect on the displacement and stress variation of the paste filling quarry. The larger the filling rate is, the smaller the stress and displacement of the overburden; the larger the filling step is, the larger the displacement and stress change of the overburden, and vice versa. The displacement curve along the direction of the rock layer basically shows "arch" type distribution characteristics, and the trend of stress change is "large-small-large" and shows "Z The trend of stress change is "large-small-large" and "Z" type distribution.

## Data Availability

All data supporting the findings in this study are available from the corresponding author upon reasonable request.
